# Age Friendly Health Systems: Pivoting from Breadth to Depth

**DOI:** 10.1177/00469580251390284

**Published:** 2025-10-31

**Authors:** Robert E. Burke, Leslie J. Pelton

**Affiliations:** 1 Divisions of General Internal Medicine and Hospital Medicine, University of Pennsylvania Perelman School of Medicine, Philadelphia, USA; 2 Center for Healthcare Evaluation, Research, and Promotion, Corporal Crescenz VA Medical Center, Philadelphia, PA, USA; 3 Leonard Davis Institute of Health Economics, University of Pennsylvania, Philadelphia, USA; 4 The John A. Hartford Foundation, New York, USA

**Keywords:** age-friendly health systems, 4Ms, age friendly care

## Abstract

The Age-Friendly Health Systems movement has demonstrated remarkable reach, with thousands of health systems now recognized as Age-Friendly. We have served as co-Editors of this Special Issue, which comes at a pivotal time in the Age-Friendly Health System movement. Published in this Special Issue are articles that meaningfully move the field forward by: (1) describing implementation and effects of Age-Friendly adoption across diverse settings of care; (2) contending with the challenge of consistent measurement of the 4Ms of Age-Friendly Care; (3) rigorously evaluating how best to implement and evaluate Age-Friendly care processes; and (4) exploring how policy levers align with Age-Friendly principles. These articles also reveal that while the Age-Friendly Movement has achieved tremendous breadth, the movement must pivot to achieve depth of clinical practice to ensure all older adults receive Age-Friendly care, and depth of research rigor to demonstrate impact and promote sustainability. To make this transition, novel tools are needed to make Age-Friendly care delivery integrated into workflows and the standard of care for older adults. In addition, alignment between payment and policy levers and Age-Friendly implementation must be expanded—including investing in higher levels of recognition that recognize depth of practice, and investment in Age-Friendly Learning Health Systems to encourage both depth of clinical practice and research rigor.

## Introduction

The Age-Friendly Health System (“AFHS”) movement—which promotes adoption of an evidence-based clinical framework called the “4Ms” (What Matters, Medication, Mentation, and Mobility)—was designed to ensure older adults received evidence-based care in all care locations even when there was no geriatrics-trained clinician. It is one of the most successfully disseminated geriatrics interventions in the history of the field, represented by the number of health systems engaged, the research undertaken, and policy and payment levers being activated. Thousands of care sites are now recognized as AFHS.

**Figure 1.**
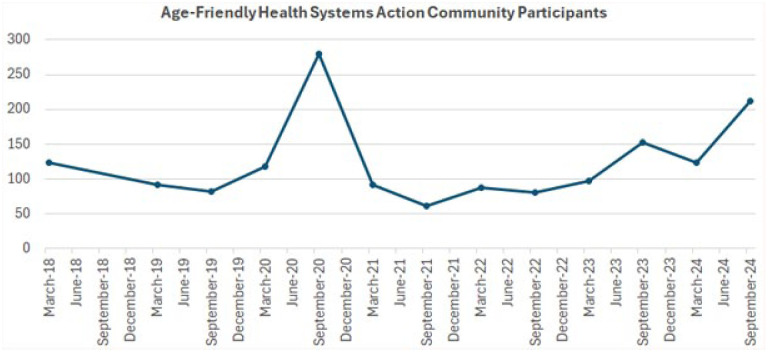
Action community participants seeking to implement age-friendly health systems. *Note.* Each time point on the *X*-axis represents the number of active Action Community participants over time; participants are usually teams from specific health care systems.

**Figure 2.**
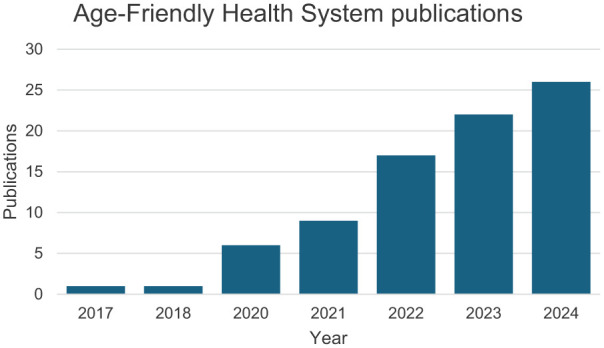
Number of academic publications regarding age-friendly health systems. *Note.* Publications with “Age-Friendly Health System” in the title or abstract were searched in PubMed to calculate the number of annual publications per year.

Action Communities, the primary mechanism for sites to be recognized as AFHS, continue to be highly subscribed (Figure 1), and academic peer-reviewed publications in AFHS are growing (Figure 2).

Serving as co-Editors on this Special Issue dedicated to Age-Friendly Health Systems has been an outstanding opportunity to reflect on the status of the field, and to chart future directions. When approached by the journal to consider this issue, we identified some a priori gaps we hoped to fill:

- Publishing higher quality and more rigorous evaluations of the effect of 4Ms care on patient outcomes, particularly for the “4Ms” practiced as a set;
- Use of implementation science-informed approaches to study and understand implementation and dissemination outcomes, to inform best practices for deep implementation that ensure 4Ms reaches more older adults in each site of care and across settings of care;
- Describing the influence of policy, regulatory, payment, and certification levers to support and sustain Age-Friendly transformation;
- Highlighting the experience of Age-Friendly transformation outside the United States, with a focus on how this differs (eg, the Institute for Healthcare Improvement is working with the government of Saudi Arabia to implement the 4Ms in the national electronic medical record).

The articles in this Special Issue move the field forward in several ways. These publications reflect hard-won experience many sites can learn from on their own Age-Friendly journeys.

A first group of articles describe implementation and effects of 4Ms adoption across diverse settings of care: inpatient hospital wards, rural primary care, telehealth for palliative care and even for-profit convenient care clinics. These articles demonstrate AFHS spread in care settings well beyond where the initial adoption of the 4Ms started—in geriatric care settings. In addition, the first systematic review of implementation of Age-Friendly in ambulatory settings published in this issue found that 7 out of 10 studies included in the review were based in ambulatory care settings that were non-geriatric specialties.^1^ If we truly want all older adults’ care to include assessment and acting on of the 4Ms, no matter where they are seen, such efforts are critical to understand and replicate at scale.

A second group of articles thoughtfully contend with the challenge of consistent measurement of assessing and acting on the 4Ms. We see in these articles essential learning about how to assess and act on each of the 4Ms as a potential initial approach to reliable practice of the 4Ms as a set. While the 4Ms are interrelated and balanced in their importance^2^ it was clear from the articles that they are not yet reliably practiced together as a set.^3^ What Matters is arguably the starting point for the delivery of Age-Friendly care^4^ but found in one of the articles to be the one of the 4Ms implemented least frequently in a hospital stay.^3^

The design of the Age-Friendly Health Systems definition allows for local adaptation and, therefore, does not lend itself to a consistently defined data set between facilities, settings of care, or health systems. In addition, while each of the 4Ms is evidence-based, the innovation of an Age-Friendly Health System is the reliable assessment and acting-on of the 4Ms as a set. One article addressed this by measuring outcomes simply related to the assessment of the 4Ms and reported that older adults whose hospital admissions included the 4Ms assessment upon admission were more likely to spend the 30 days post-discharge at home than those older adults whose care was not Age-Friendly. This effect held for older adults whose assessment upon admission only included three of the 4Ms.^5^ Other articles offer a composite measure, which ensures capturing the 4Ms assessment and act on as a set as a starting point for other health systems^6^ and recommendations for 4Ms implementers, clinicians, and researchers to advance the evidence of 4Ms on health outcomes.^7^ These articles also point to other relevant gaps, such as the inclusion of unpaid caregivers into AFHS measurement. For example, results in 1 article identify the suggestion from older adults to engage family caregivers in eliciting What Matters as part of 4Ms care.^4^ The inclusion of family caregivers in these articles, and the development of My Health Checklist (My Health Checklist, Boston, MA, USA, Institute for Healthcare Improvement, 2025, www.IHI.org)—designed in part for family caregivers to prepare with older adults for their care to include the 4Ms—suggests the opportunity to further understand the role of family caregivers in the delivery of the 4Ms and older adults’ health outcomes.

A third group of articles moves the field forward in providing examples of how to improve the rigor of our research on how to implement and evaluate the effect of Age-Friendly care processes. For example, the Special Issue includes articles that rigorously describe implementation processes for interventions that focus on one of the 4Ms (and address all 4Ms): (1) testing the usability of an inpatient What Matters assessment and intervention tool with patients (called Patient Priorities Care); (2) describing the experience implementing a community-based Mentation intervention for persons living with dementia and caregivers (the Tailored Activities Program); (3) and identifying organizational characteristics are associated with the successful implementation of an inpatient predominantly Mobility intervention (STRIDE). The Special Issue also includes to our knowledge the first systematic review of implementation strategies used for Age-Friendly adoption.^1^

Last, this Special Issue includes some of the first articles exploring how policy levers (such as Bundled Payments for Care Improvement^5^ and the new CMS Hospital Age-Friendly Measure^8^ align with Age-Friendly Health Systems delivery and outcomes. One article also demonstrates how 4Ms training can advance quality metrics that affect payment, in this case the Merit-Based Incentive Payment Systems (MIPS) Measures, part of the Medicare incentive system.^9^

**Figure 3.**
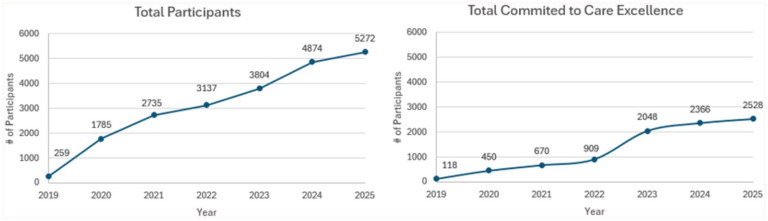
Number of age-friendly health system recognized sites by type of recognition. *Note.* Total Age-Friendly Health System participants per year recognized at a Level 1 are portrayed in the left side over time. On the right side, total age-Friendly Health System participants recognized at a Level 2 per year are displayed.

Together, what do these studies reveal about where we are at this point on the Age-Friendly research journey? If we were to write this same article in a year, what would we want to see? We see that these studies reveal tremendous breadth, but we believe the field must continue to pivot to depth in 2 key ways: depth of rigor and depth of practice. In terms of depth of rigor, we have very few prospective controlled studies (such as hybrid implementation-effectiveness trials^10^) or retrospective observational studies employing methods that generate causal inferences about the effect of the 4Ms as a set on outcomes. Most studies in the field are focused on describing implementation, rather than comparing strategies to optimize implementation or demonstrate the effect of 4Ms practice on patient outcomes. In terms of depth of practice, it’s clear from these publications that reliable and systematic practice of the 4Ms across care settings—and mechanisms to use the 4Ms in a longitudinal way—is still very difficult for most health systems. This struggle is reflected in the overall patterns of Age-Friendly recognition across the country over time (Figure 3), showing continued spread of Level 1 recognition with a significant lag to Level 2 recognition, and the marked variability in uptake in different care settings.

How, then, do we get from breadth to depth? First, we must focus on continuing to make it easier to do the right thing: to provide 4Ms care at every clinical encounter with older adults. More inquiry is needed into how best to embed automated tools in the EHR, deploy digital health strategies and increasingly sophisticated predictive algorithms, and even use large language models to reliably and systematically deliver 4Ms care at scale in an integrated way. Second, we must continue to build alignment between payment and policy levers and Age-Friendly implementation. The CMS Age-Friendly Hospital Measure^8^ has raised the awareness of many health systems about the need to engage with Age-Friendly implementation and measurement. It represents a critical first step directly linking payment to Age-Friendly practices, and there is much to be learned about its impact as a first step—a simple attestation measure—and to what extent additional measurements and incentives are needed to manifest real depth of change. While health systems will continue to be tasked to accomplish this work at a local and system level, we recognize that the onus for sustaining the 4Ms movement and ensuring it becomes the new care paradigm for all older adults must move beyond health systems to national regulatory and payment frameworks.^11^

Along these lines, payers should invest in recognition that are tied to depth of 4Ms adoption. Level 1 Age-Friendly Recognition requires an approved plan for assessing and acting on the 4Ms, and Level 2 requires auditing performance to ensure the 4Ms are being assessed and acted on in a single clinical setting within a larger health system. These low barriers to entry promote spread, but not depth of practice. The Institute for Healthcare Improvement’s current System-Wide Spread Collaborative is an important example to follow. Thirty health care systems that have achieved Level 2 recognition in at least one care setting are working towards spread of 4Ms care to additional care settings in the Collaborative, reaching more than half of their older adults with 4Ms care, and report on outcomes. We would argue this represents “Level 3” recognition that promotes depth of practice. Alongside this work, UCSF Age-Friendly Health Systems Research Council is developing a maturity survey to assess the depth of 4Ms adoption a health system achieves. This maturity scale creates the opportunity to characterize 4Ms depth of practice and its impact on health outcomes of older adults and family caregivers.

Payers could incentivize this transformation to depth by augmenting billing for 4Ms care commensurate with each level of recognition. An analogous payment structure already exists in the Patient-Centered Medical Home model, combining increased fee-for-service payments for higher levels of recognition with additional per-member per-month payments to coordinate care.^12^ As more of the Medicare population moves into Medicare Advantage plans, there is also an opportunity to understand their role in increasing 4Ms adoption. The Institute for Healthcare Improvement is currently working with 4 Medicare Advantage plans to identify levers for increased adoption of the 4Ms, and to understand the financial impact and assess the business case. Further research is needed on whether, and which, payment levers expand 4Ms reach.

Finally, depth of practice will be best achieved within structures that promote continued improvement in 4Ms practice that drives continued improvement in patient outcomes on an urgent timeline—the U.S. population is aging now and needs this care. We believe an Age-Friendly Learning Health System model provides an opportunity to pair effective implementation and quality improvement practice with rigorous evaluation of outcomes.^13^

This Special Issue comes at a pivotal time in the Age-Friendly Health System movement. Having achieved remarkable spread, the model must pivot to depth of practice to ensure all older adults receive Age-Friendly care, and depth of rigor to demonstrate impact and promote sustainability. Bringing together providers, payers, and researchers in a Learning Health System model supported by national regulatory and payment incentives holds the most promise for achieving transformational change.
